# Bridging the gap between informatics and medicine upon medical school entry: Implementing a course on the Applicative Use of ICT

**DOI:** 10.1371/journal.pone.0194194

**Published:** 2018-04-23

**Authors:** Natasa M. Milic, Nikola Ilic, Dejana M. Stanisavljevic, Andja M. Cirkovic, Jelena S. Milin, Zoran M. Bukumiric, Nikola V. Milic, Marko D. Savic, Sara M. Ristic, Goran Z. Trajkovic

**Affiliations:** 1 Department for Medical Statistics and Informatics, Faculty of Medicine, University of Belgrade, Belgrade, Serbia; 2 Department for Internal Medicine, Mayo Clinic, Rochester, United States of America; 3 Center for Information and Communication Technologies, Faculty of Medicine, University of Belgrade, Belgrade, Serbia; 4 Faculty of Medicine, University of Belgrade, Belgrade, Serbia; 5 Faculty for Media and Communications, Singidunum University, Belgrade, Serbia; University of Gondar, ETHIOPIA

## Abstract

Education is undergoing profound changes due to permanent technological innovations. This paper reports the results of a pilot study aimed at developing, implementing and evaluating the course, "Applicative Use of Information and Communication Technologies (ICT) in Medicine," upon medical school entry. The Faculty of Medicine, University of Belgrade, introduced a curriculum reform in 2014 that included the implementation of the course, “Applicative Use of ICT in Medicine” for first year medical students. The course was designed using a blended learning format to introduce the concepts of Web-based learning environments. Data regarding student knowledge, use and attitudes towards ICT were prospectively collected for the classes of 2015/16 and 2016/17. The teaching approach was supported by multimedia didactic materials using Moodle LMS. The overall quality of the course was also assessed. The five level Likert scale was used to measure attitudes related to ICT. In total, 1110 students were assessed upon medical school entry. A small number of students (19%) had previous experience with e-learning. Students were largely in agreement that informatics is needed in medical education, and that it is also useful for doctors (4.1±1.0 and 4.1±0.9, respectively). Ability in informatics and use of the Internet in education in the adjusted multivariate regression model were significantly associated with positive student attitudes toward ICT. More than 80% of students stated that they had learned to evaluate medical information and would use the Internet to search medical literature as an additional source for education. The majority of students (77%) agreed that a blended learning approach facilitates access to learning materials and enables time independent learning (72%). Implementing the blended learning course, "Applicative Use of ICT in Medicine," may bridge the gap between medicine and informatics upon medical school entry. Students displayed positive attitudes towards using ICT and gained adequate skills necessary to function effectively in an information-rich environment.

## Introduction

Education has undergone profound changes due to recent technological advancements [1–3]. Information and Communication Technologies (ICT) resources have become available, both for support of on-site education and for on-line learning. It is both important and interesting to assess the knowledge acquired and attitudes that students have towards their use [4–6]. Mobile and portable devices with wireless broadband access, such as smartphones and tablet computers, in recent years have been widely used in student populations. Mobile technologies have enabled a novel form of learning whereby students have immediate and round the clock access to educational materials [7–10].

Students’ learning success is highly dependent on their skills involving ICT use, particularly in medicine [11, 12]. The need to be familiar with a constantly evolving body of information to meet everyday professional demands is an ongoing challenge. Constant change is the new normal. The concept of lifelong learning [13], as an ongoing pursuit of knowledge which enhances personal development, but also self-sustainability, competitiveness and employability, have become of vital importance in medicine. The introduction of new technologies has changed how we receive and gather information, collaborate with others and communicate, while assistive technologies have become important considerations when discussing lifelong learning.

Traditional methods of learning are time intensive, for both teachers and students, thus innovative methods are being introduced in medical education to optimize time management. Blended learning courses, oriented towards more personalized learning styles, are being implemented in medical school curricula [1, 14, 15]. Certain skills are needed for quality assessment of medical information gained in everyday work, and skills for gathering medical information from specialized electronic databases are prerequisites for physicians today. Access to these modern learning opportunities, however, is dependent upon student skill in using these high tech assistive technologies. There is great potential to support learning with the emergence of Web 2.0 technologies, which have provided students with several means by which they can access and assimilate information in an informal manner, on a daily basis [16, 17]. Universities and students need to acquire sufficient flexibility in order to remain current and relevant in a rapidly changing educational environment. This suggests that the greatest accomplishment of any university is to teach its students how to learn. A thorough understanding of Web 2.0, in addition, is also a necessity in the constantly changing information world. We aimed in this study to map and bridge the gaps between the existing possibilities that modern ICT offer and their applicative uses in the context of Serbian medical education. The goal of the study was to assess knowledge, use, and student attitudes towards ICT among first year medical students, to explore factors related to student attitudes towards the use of ICT in medical education, and to develop, implement, and evaluate the blended learning course, “Applicative Use of ICT in Medicine," upon medical school entry.

## Methods

**Fig 1:**
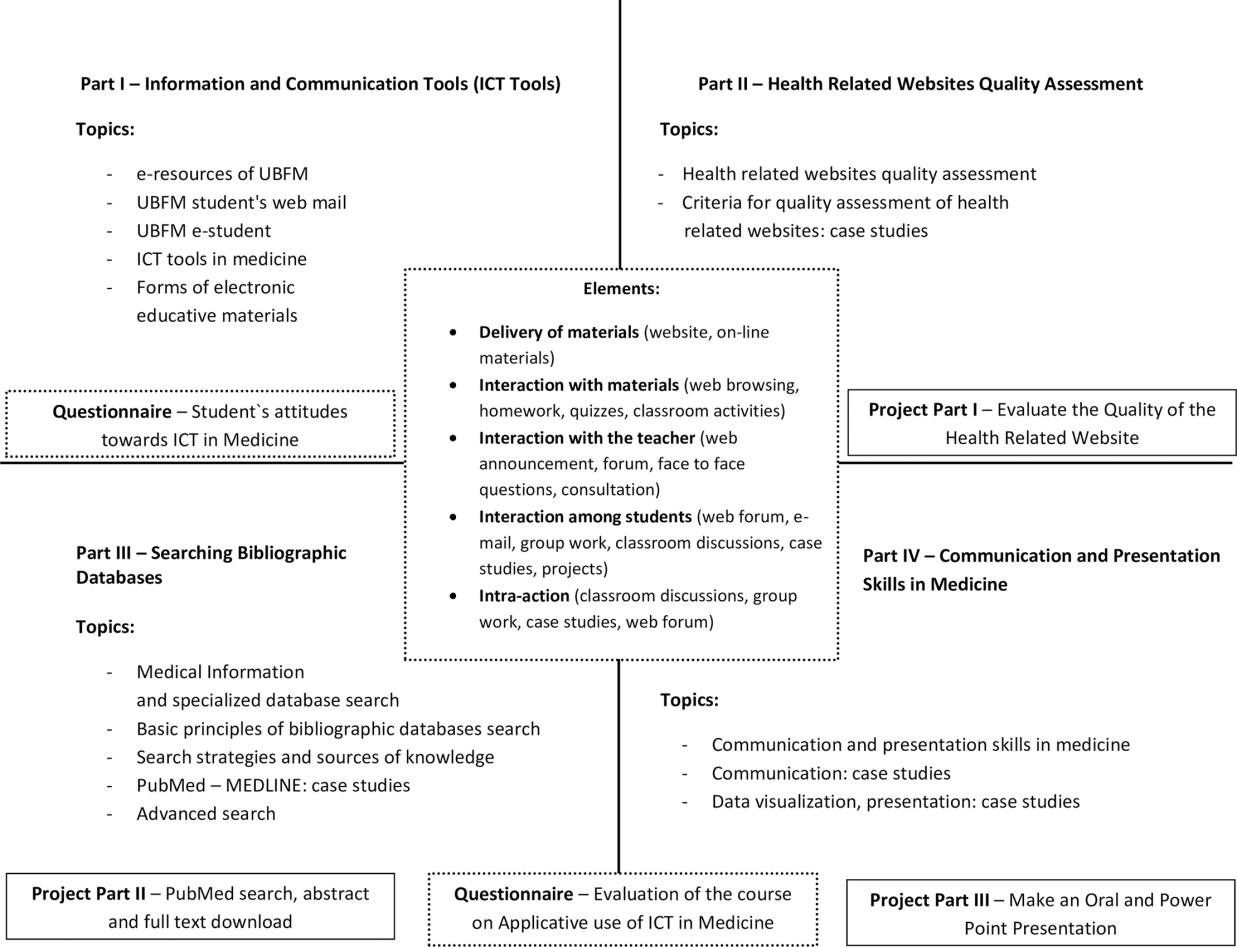
Structure of the blended learning course, Applicative Use of ICT in Medicine” (legend: UBFM—University of Belgrade, Faculty of Medicine).

This was a prospective cohort study conducted with first year medical students attending the Faculty of Medicine, University of Belgrade (UBFM). The two cohorts consisted of all medical students enrolled at UBFM undergraduate program during 2015/16 (n = 570) and 2016/17 (n = 540) academic school years. Only students not registered for course were excluded from study (n = 17). The UBFM is one of the largest medical schools in Europe, with more than 500 students enrolled each year. It is a public institution founded a century ago, and since its inception, it has been the main source of Serbian medical professionals and scholars. A major curriculum reform was introduced at UBFM in 2014 which consisted of several novel elements, one of which included the implementation of the course, “Applicative use of ICT in Medicine,” upon medical school entry. The obligatory, semester long course was implemented during the first semester of medical studies, with a total of 30 curriculum hours. The course was designed using a blended learning format to introduce the concepts of a Web-based learning environment to medical students at the beginning of their professional education. The content of the course was developed using established principles of curriculum development. It included: 8 lectures, 16 hours of practical class work and 6 hours of seminars covering four modules: Information and Communication Tools, Health Related Websites Quality Assessment, Bibliographic Databases Search, and Communication and Presentation Skills in Medicine. The course included structured live group activities and case discussions, in addition to formal lectures. The teaching approach was supported by the multimedia didactic materials which the students studied by computer via the Internet, using the Moodle Learning Management System. Students had the option of posting questions through a web portal to facilitate discussions with fellow students and course faculty. The elements of the course are provided in detail in Fig 1.

A structured questionnaire designed by faculty members was administered to students to map their knowledge, use and attitudes towards ICT at the beginning of the course. The questionnaire was designed after an extensive review of the literature, interviews with students and teachers, and it was pretested on the medical school class of 2014/15 (n = 550). It included: 1) basic demographic data, 2) questions related to student self-assessed knowledge in informatics and computers, 3) questions related to student use of ICT and 4) questions related to student attitudes towards ICT. Students were asked to rate their knowledge on a five-point Likert scale (from 1 = very poor to 5 = very good). High scores indicated that a student had a high self-perception of his/her overall knowledge in informatics and computers.

Items related to student use of ICT included questions about having Internet access, daily duration of Internet use, social network, tablet, and smartphone use, as well as Internet use for information research and/or education. Attitudes towards ICT were determined by student agreement or disagreement with statements about the importance of ICT in medical education, clinical practice and everyday life. A five level Likert scale was used to measure items related to student attitudes towards ICT. These attitudes were assessed using the average score for each question. Mean scores above neutral were considered as positive attitudes.

Evaluation of the course included objective as well as subjective components. The formal (objective) evaluation of student achievement was measured by a final score which integrated all course activities throughout the semester. The final score was calculated by summing the knowledge test score (composed of several tests on each topic) and weighted 0.6, and individual project activities (weighted 0.4), which included three problem-based components (weighted 0.1, 0.15 and 0.15). Final scores ranged from 0 to 100, with 80 points or more suggesting achievement of student competence in, the “Applicative use of ICT in Medicine” course. An anonymous course evaluation consisting of a questionnaire designed by faculty members was distributed to students at the end of the course. Responses for each item were ranked from 1 (strongly disagree) through 3 (neutral) to 5 (strongly agree), using the 5-point Likert method. Those with scores above neutral were labeled as positive, while those below neutral were labeled as negative. The two statements, “Medical students need informatics” and “Informatics is useful for doctors” were part of both the pre-course questionnaire, as well as the one distributed after course completion. Appearance of the Questionnaires as well as the Certificate of course completion in the Moodle virtual learning environment is shown in S1, S2 and S3 Figs.

Ethical approval for the study was obtained from the Institutional Review Board (IRB) of the University of Belgrade, Faculty of Medicine. The purpose of the study was explained to the students and oral consent was obtained and documented in their records at the beginning of the course. The IRB approved the use of oral consent as there was no potential harm to the study participants.

### Statistical analysis

Descriptive statistics on student demographic characteristics, numerical questionnaire responses (Likert scale) and final scores are reported as mean with standard deviation. Data distribution was assessed visually (based on graphs) and by using the common descriptive statistics including, mean, standard error and skewness coefficient. Categorical data are presented as numbers with percentages. Cronbach’s alpha coefficient was used to assess the reliability of the surveys. Differences between the two student classes were analyzed using Student’s t test for numeric variables, and the Pearson chi-squared test for categorical variables. Differences between paired data were analyzed using the paired samples t test. The univariate and multivariate logistic regressions were used to determine the independent predictors for positive student attitudes toward the use of ICT in medicine. Multiple regressions were conducted in a stepwise manner, using the Ordinary Least Squares method [18]. This approach allowed for the selection of a limited set of statistically significant predictors from variables found to be significant by earlier analyses. Results were expressed as *B*, Wald Chi-Square, odds ratios (OR), and their 95% confidence intervals (CI). All tests were two-tailed. Data met the assumptions for each statistical test applied. P<0.05 was considered statistically significant. All analyses were conducted using the Statistical Package for the Social Sciences (IBM SPSS, version 21).

## Results

A total of 1110 students took the course during the first academic semesters of 2015 and 2016. Mean student age was 18.95±0.88 years, and most participants were female (69%), mirroring the predominance of female sex in the total medical school population at our institution. There were no statistically significant differences in age and sex between the 2015 and 2016 cohorts (p = 0.718 and p = 0.580, respectively). The questionnaire evaluating knowledge, use and attitudes towards ICT, as well as the anonymous program evaluation, had a high return rate (>90% of the student population completed both). Student demographic data and response rates per academic school year are shown in Table 1. Analysis of internal consistency showed that the Cronbach’s alpha was 0.77 for the first and 0.83 for the second questionnaire, indicating good scales reliability.

**Table 1:** Student demographic data and response rates per academic school year.

Item	Total	2015/16	2016/17
Total number of registered students	1110	570	540
Age, mean ± sd*	18.95±0.88	18.94±0.88	18.96±0.87
Female, n (%)**	768 (69.2%)	391 (68.6%)	377 (69.8%)
Response rate first questionnaire	1012	557	455
Response rate second questionnaire	1015	476	539

* for comparison between student class years, t test was applied

** for comparison between student class years, Chi square test was applied

A small number of students (18.6%) had previous experience with e-learning. Participants reported abilities above neutral in informatics (3.67±0.89) and computers (3.87±0.89). No significant differences were found between students from the two class years regarding their abilities in informatics and computers (Table 2).

**Table 2:** Self-reported ability in informatics and computers for two student classes upon medical school entry.

Item	Total	2015/16	2016/17	p*
Ability in informatics, $\bar{x}\pm sd$	3.67±0.90	3.66±0.89	3.68±0.90	0.633
Ability in computers, $\bar{x}\pm sd$	3.86±0.89	3.85±0.88	3.88±0.90	0.543

* t test was applied

Of the 1010 respondents, 981 (96.9%) of them searched the Internet for educational content, and 948 (93.7%) of them had home Internet access (Table 3). The majority of students used Smartphones for educational information searches via the Internet (90.5%). Students from the 2016/17 class used smartphones significantly more often than the 2015/16 students for e-mail, education information searches, and sharing notes (85.3% vs 74.2%, 93.0% vs 88.5%, 65.1% vs 55.6%, respectively).

**Table 3:** Student distribution according to Applicative Use of ICT for two student classes upon medical school entry.

Item	Total,	2015/16	2016/17	p*
	n (%)	n (%)	n (%)	
Internet use for information research	981 (96.9)	540 (96.9)	441 (96.9)	0.982
Have Internet access from home	948 (93.7)	527 (94.6)	421 (92.5)	0.175
Social network use	966 (95.5)	527 (94.6)	439 (96.5)	0.156
Daily use of internet ≤4 hours	853(84.0)	478 (85.8)	372 (81.7)	0.096
Tablet use	321(31.7)	181 (32.5)	140 (30.8)	0.557
Smartphone use	969(95.8)	530 (95.3)	439 (96.5)	0.358
e-mail	800 (79.2)	412 (74.2)	388 (85.3)	**<0.001**
watch lectures	270 (26.8)	149 (26.8)	121 (26.6)	0.999
education information search	914 (90.5)	491 (88.5)	423 (93.0)	**0.015**
read lecture notes	455 (45.1)	237 (42.8)	218 (47.9)	0.103
share notes	605 (59.8)	309 (55.6)	296 (65.1)	**0.002**

* Chi square test was applied

Students overall reported positive attitudes towards applying ICT in medicine (Table 4). A majority of the students agreed with statements that medical students need informatics education (4.05±0.95), and also that medical informatics is useful for doctors (4.14±0.92). Most positive student attitudes related to the statement that, “Computers make everyday life easier” (4.37±0.89). There was only one significant difference between the cohorts in attitudes, with students from the class of 2016/17 more strongly agreeing with the statement that: “Internet is needed during schooling” (4.36±0.87 vs 4.16±0.94).

**Table 4:** Student attitudes towards ICT at the beginning of the course.

Item	Total	2015/16	2016/17	p*
	x ± sd	x ± sd	x ± sd	
Medical students need Informatics	4.04±0.96	3.99±0.98	4.09±0.93	0.093
Informatics is useful for doctors	4.13±0.93	4.09±0.94	4.18±0.91	0.108
Internet is needed during education	4.24±0.92	4.15±0.94	4.36±0.87	**<0.001**
Computers make everyday life easier	4.37±0.90	4.34±0.90	4.41±0.89	0.184

* t test was applied

Self-rating of ability in computers and use of the Internet in education, in a multivariate regression model, was significantly associated with student attitudes toward ICT after adjusting for age and gender (Table 5). Medical students who searched the Internet for information research and who indicated a better self-rating of their computer competency demonstrated more positive attitudes towards the need for education in informatics compared to those who did not use the Internet, and who reported a poor self-rating of their abilities in using computers.

**Table 5:** Logistic regression analysis of positive student attitudes towards, “Applicative Use of ICT in medicine,” as a function of knowledge, use, and attitudes towards ICT upon medical school entry (both class years).

				95% Confidence Interval for Odds Ratio	
Variables	*B*	Wald Chi-Square	Odds Ratio	Lower	Upper
Ability in informatics	0.503	31.804	1.654	1.389	1.971
Computers make everyday life easier	0.541	42.081	1.718	1.459	2.023
Internet use for educational research	1.375	10.883	3.957	1.748	8.958
(Constant)	-4.400	56.644			

Among the students who responded to the second questionnaire (n = 1015), 82% stated that the course was simple and user-friendly, 80% stated that medical students need informatics and 83% of students stated that informatics is useful for doctors (Table 6). The mean scores for the statements, “Medical students need Informatics” (4.17±0.92) and “Informatics is useful for doctors” (4.23±0.85) were significantly higher compared to the scores obtained from the first survey (p<0.001 and p = 0.001, respectively). The majority of students (66%) enjoyed the course, 77% of them agreed that using a blended learning approach facilitated access to learning materials, and enabled time independent learning (72%). 83% of students stated that during the course, they learned how to evaluate medical information and that Internet search of medical literature will become an additional source of knowledge in their future education.

**Table 6:** Evaluation of the course–both cohorts.

Evaluation of the course	Negative	Neutral	Positive
	n (%)	n (%)	n (%)
Medical students need Informatics	49 (4.8%)	156 (15.4%)	800 (79.8%)
Informatics is useful for doctors	37 (3.6%)	134 (13.2%)	844 (83.2%)
Facilitates access to learning materials	63 (6.2%)	174 (17.1%)	778 (76.7%)
Enables time independent learning	96 (9.5%)	188 (18.5%)	731 (72.0%)
It was funny	123 (12.1%)	225 (22.2%)	667 (65.7%)
It was too detailed	735 (72.4%)	212 (20.9%)	68 (6.7%)
Encourages a new way of thinking	205 (20.2%)	410 (40.4%)	400 (39.4%)
Difficult subject matter	843 (83.1%)	103 (10.1%)	69 (6.8%)
Simple and user-friendly	64 (6.3%)	116 (11.4%)	835 (82.3%)
Would choose another on-line course	136 (13.8%)	298 (30.1%)	555 (56.1%)
Learned how to evaluate medical information	37 (3.6%)	129 (12.7%)	849 (83.7%)
Internet search of medical literature becomes an additional source of knowledge	43 (4.2%)	128 (12.6%)	834 (83.2%)
Communication technologies in medicine are important	64 (6.3%)	179 (17.6%)	792 (76.1%)
Satisfied with the knowledge gained	58 (5.7%)	196 (19.3%)	761 (75.0%)
Had technical difficulties	687(67.7%)	145 (14.3%)	183 (18.0%)
Would recommend on-line learning to others	107 (10.5%)	304 (30.0%)	624 (59.5%)

## Discussion

This study sets the stage for shaping the efforts of medical educators to bridge the gap between the growing demands of expanding medical school curricula and student premedical school skills in ICT. The paper presents the results of the implementation of the blended learning course, “Applicative Use of ICT in Medicine,” upon medical school entry. The main finding of this study was that students held overall positive attitudes towards ICT and the course received positive evaluations by students. The results of the study imply that students will continue to further their use of ICT in their medical education and future practice.

The University of Belgrade is a leading institution of higher education in the region in large part by adapting to the challenges of modern times, while preserving worthwhile aspects of tradition, and this is particularly true in medicine. Traditional learning methods should not be compromised; however, the rapid pace at which medicine is evolving necessitates incorporating novel technologies into the educational environment. Student ability in informatics is an important factor for the further development of skills needed for optimizing cooperation, communication, problem solving and lifelong learning [19, 20]. Recent studies suggest that introducing ICT into education in developing countries has many barriers, such as the availability of equipment, accessories and proper maintenance. However, overcoming these barriers would be a major step in promoting the need for involving ICT in medical education [21, 22]. Recently, the UBFM implemented a curricular reform which included the implementation of the, “Applicative Use of ICT in Medicine,” course upon medical school entry. The medical program at UBFM is of 6 year duration, covering basic, clinical and public health sciences courses, summer rotations for clinical practice and internships. Students enroll in medical school just after high school completion, with very different levels of knowledge and skills in informatics upon medical school entry. Most high schools include components of informatics in their curricula, but they are focused largely on tools of productivity (Microsoft Office use, email etc.). Other, informal ways by which they gain skills in informatics before medical school entry include: use of smartphones, tablets, social networks etc. A growing majority of students currently use mobile devices for learning and access to information. Student attitudes regarding mobile learning generally have been positive [23, 24]. Uzunboylu, Cavus, and Ercag [25] reported that both students and instructors liked using mobile devices for learning. In our study, first year medical students used smartphones more often than tablets. Smartphones mostly were used for information searches (97%), rather than for formal ways of learning, such as reading (45%) and listening to lectures (27%). A similar study conducted in Japan reported infrequent use of smartphones for formal learning due to the small screen, difficult information input, and use in an environment that is often noisy and distracting [26].

The introduction of the course, “Applicative Use of ICT in Medicine,” aimed to bridge the gap between previous different levels of knowledge and use of ICT upon medical school entry and ICT practical applications in medicine. Student acquisition of competences that will facilitate learning and practicing medicine was shown to be of special interest upon medical school entry. An understanding of ICT tools critical for students to learn the selective and proper use of information available from vast online resources was the major goal of the course. Innovation in science and technology has had a profound effect on learning. Learning has expanded beyond the traditional classroom methods such that knowledge can be acquired and applied regardless of place or time. Therefore, the UBFM course was designed in a blended learning format to incorporate traditional and more modern means of imparting information. The “Applicative Use of ICT in Medicine” was introduced into the medical school curriculum as an accredited and obligatory course. This was deemed necessary to relay the importance and legitimacy of informatics in medicine to both students and faculty, and we believe that the formalization of the course was crucial for its successful implementation.

Students generally were very positive towards the need for informatics, both for medical students and doctors. According to the recommendations of the International Medical Informatics Association (IMIA), it is necessary, not only for IT specialists, but also equally important for students of medicine, bioinformatics, biomedical engineering, molecular biology, chemistry, public health workers and clinicians, to become familiar with ICT educational tools used in their learning and/or practice environments. This may be achievable through undergraduate or postgraduate studies [27]. Other studies largely have reported similar results on this topic [28, 29]. A few authors have reported a predominantly neutral attitude on the part of students towards informatics in medicine [30].

Students in our study self-reported abilities in informatics that were above neutral, and this was predictive of positive student attitudes towards ICT use in medicine. In a multivariate regression model, better self-reported ability in informatics and more use of the Internet for information searches for educational purposes were key predictors of positive student attitudes towards the applicative use of ICT. The same conclusion regarding the knowledge of information technologies and Internet searches was reported by Houshyari et all [30]. In the same study, the mean score of self-assessed computer knowledge was higher for males than for females, but there was no relationship between attitudes towards ICT and gender. Previous experience with e-courses influences positive attitude towards e-learning according to a study by Brumini [31], but this was not confirmed by our findings. According to our study results, students’ previous experiences in web-based courses were very limited. Only 19% of the first year students had any experience with on-line courses during their pre-medical education. It was shown that students without previous experience had less knowledge, but very positive attitudes towards the necessity of on-line and computer courses [32]. Introducing students to a blended learning course on, “Applicative Use of ICT in Medicine,” at the very beginning of their medical education seemed to be very useful for the subsequent application of ICT within the context of Serbian medical education.

There was positive feedback from medical students regarding the course. High scores were recorded from the evaluation questionnaire for the questions and statements related to student attitudes towards ICT. The greatest success was the general satisfaction with the course. The vast majority was pleased with the quality of knowledge to which they were exposed, and the means by which the knowledge was obtained.

The work presented here has some limitations. The results are from a single public medical school in Serbia, which can limit the generalizability of the findings to other settings. It describes the introductory course in the first year of implementation of a new curriculum that partially covers the generic competences in biomedical informatics that medical students should acquire. Further implementation of courses covering all generic competences that must be achieved after graduation, as suggested by the IMIA, should be the final goal. The introduction of new obligatory learning formats to medical studies, is the result of the work of a collaborative group with extensive experience in developing blended learning modules, and may not reflect the situations of educators with less experience. Formal summative tests were performed in order to objectively assess gained knowledge and skills, but pre- and post- test evaluations were not conducted.

## Conclusion

This study indicated that implementing the course, “Applicative Use of ICT in Medicine,” may bridge the gap between medicine and informatics at the time of medical school entry. Students displayed positive attitudes toward using ICT and were satisfied with the knowledge and skills that were gained, which are necessary to function effectively in an information-rich environment.

## Supporting information

S1 Fig Appearance of the questionnaire regarding knowledge, use and attitudes towards ICT distributed to students at the beginning of the course “Applicative Use of ICT in medicine” in the Moodle virtual learning environment. (PDF)

S2 Fig Appearance of the anonymous course evaluation questionnaire distributed to students at the end of the course “Applicative Use of ICT in Medicine” in the Moodle virtual learning environment. (PDF)

S3 Fig Certificate of completion. (PDF)

S1 File Minimal dataset underlying research findings (.csv format). (CSV)
