# Supplementary material for: Bridging the gap between informatics and medicine upon medical school entry: Implementing a course on the Applicative Use of ICT
Source: PLoS One. 2018 Apr 23;13(4):e0194194. doi: 10.1371/journal.pone.0194194 (PMC5912767; doi:10.1371/journal.pone.0194194)
Supplement: S1 Fig — (PDF) [file pone.0194194.s001.pdf]

S1 Fig. Appearance of the Questionnaire regarding knowledge, use and attitudes towards ICT distributed to students at the beginning of the course “Applicative Use of ICT in Medicine” in the Moodle virtual learning environment.

Please read and answer on every question presented bellow.

Question #1

Gender:

○ Male ○ Female

Question #2

Age, yrs:

[ ]

Question #3

Previous education:

Izaberi...

Question #4

Previous experience with e-learning?

○ Yes ○ No

Question #4

For next questions/statements please mark one out of 5 possible levels of agreement, from **1 (lowest grade/strong disagreement)** through **3 (neutral)** to **5 (highest grade/strong agreement)**.

		1	2	3	4	5
How would you grade your ability in informatics?	◉	○	○	○	○	○
How would you grade your ability in computers?	◉	○	○	○	○	○
Medical students need informatics.	◉	○	○	○	○	○
Informatics is useful for doctors.	◉	○	○	○	○	○
Internet is needed during education.	◉	○	○	○	○	○
Computers make everyday life easier.	◉	○	○	○	○	○

Question #5

Do you have internet access from home?

○ Yes ○ No

Question #6

How much time do you spent using internet daily?

○ <2 hours ○ 2-4 hours ○ 4-6 hours ○ >6 hours

Question #7

Do you use internet for information search?

- [ ] Yes
- [ ] No

Question #8

Do you use social networks?

- [ ] Yes
- [ ] No

Question #9

Do you use tablet?

- [ ] Yes
- [ ] No

Question #10

Do you use smartphone?

- [ ] Yes
- [ ] No

Question #11

Do you use smartphone for email?

- [ ] Yes
- [ ] No

Question #12

Do you use smartphone to watch lectures?

- [ ] Yes
- [ ] No

Question #13

Do you use smartphone for education_information search?

- [ ] Yes
- [ ] No

Question #14

Do you use smartphone to read lecture notes?

- [ ] Yes
- [ ] No

Question #15

Do you use smartphone to share notes?

- [ ] Yes
- [ ] No

Submit preview
