# Supplementary material for: Bridging the gap between informatics and medicine upon medical school entry: Implementing a course on the Applicative Use of ICT
Source: PLoS One. 2018 Apr 23;13(4):e0194194. doi: 10.1371/journal.pone.0194194 (PMC5912767; doi:10.1371/journal.pone.0194194)
Supplement: S2 Fig — (PDF) [file pone.0194194.s002.pdf]

S2 Fig. Appearance of the anonymous course evaluation questionnaire distributed to students at the end of the course "Applicative Use of ICT in Medicine" in the Moodle virtual learning environment.

Please read and answer every question presented bellow.

Question #1

For next statements please mark one out of 5 possible levels of agreement, from **1 (strong disagreement)** through **3 (neutral)** to **5 (strong agreement)**.

	1	2	3	4	5
Medical students need informatics.					
Informatics is useful for doctors.					
This way of learning facilitates access to learning materials.					
This way of learning enables time independent learning.					
It was funny to learn like this.					

Question #2

For next statements please mark one out of 5 possible levels of agreement, from **1 (strong disagreement)** through **3 (neutral)** to **5 (strong agreement)**.

	1	2	3	4	5
This course was too detailed.					
This course encourages a new way of thinking.					
Course was simple and user friendly.					
Would choose another course like this.					

Question #3

For next statements please mark one out of 5 possible levels of agreement, from **1 (strong disagreement)** through **3 (neutral)** to **5 (strong agreement)**.

	1	2	3	4	5
During this course I've learned how to evaluate medical information.					
Internet search of medical literature becomes an additional source of knowledge after this course completion.					
I'm aware why communication technologies in medicine are important.					
I'm satisfied with the knowledge gained.					
I had technical difficulties during this course.					
Would recommend this way of learning to others.					

Submit preview
