# Supplementary material for: Bridging the gap between informatics and medicine upon medical school entry: Implementing a course on the Applicative Use of ICT
Source: PLoS One. 2018 Apr 23;13(4):e0194194. doi: 10.1371/journal.pone.0194194 (PMC5912767; doi:10.1371/journal.pone.0194194)
Supplement: S3 Fig — (PDF) [file pone.0194194.s003.pdf]

УНИВЕРЗИТЕТ У БЕОГРАДУ
МЕДИЦИНСКИ ФАКУЛТЕТ

UNIVERSITY OF BELGRADE
SCHOOL OF MEDICINE

КАТЕДРА ЗА МЕДИЦИНСКУ СТАТИСТИКУ И ИНФОРМАТИКУ

издаје

П О Т В Р Д У

да је

Марко Марковић

број индекса: МД15····

присуствовао-ла настави и положио-ла тест провере знања на предмету под називом

ПРИМЕНА ИНФОРМАЦИОНО-КОМУНИКАЦИОНИХ ТЕХНОЛОГИЈА У МЕДИЦИНИ

Београд, 15. децембар 2016. године

руководилац предмета

Доц. др Наташа Милић

шеф катедре

Доц.др Дејана Станисављевић

БРОЈ ЕСПБ: 2
